# Childhood Posterior Reversible Encephalopathy Syndrome (PRES) in Resource Limited Settings: Addressing Diagnostic and Therapeutic Hurdles-A Case Report

**DOI:** 10.1155/crpe/9444554

**Published:** 2025-03-27

**Authors:** Bipesh Kumar Shah, Sadmarg Thakur, Prajjwol Luitel, Roshan Gaire

**Affiliations:** ^1^Department of Pediatrics, School of Public Health and Community Medicine, BP Koirala Institute of Health Sciences, Dharan, Sunsari, Nepal; ^2^Tribhuvan University Teaching Hospital, Kathmandu, Bagmati, Nepal

**Keywords:** case report, complications, imaging, parieto-occipital, seizures

## Abstract

Posterior reversible encephalopathy syndrome (PRES) is a condition that manifests with symptoms like altered mental status, seizures, vision impairment, and vasogenic edema primarily affecting the occipital and parietal lobes, with occasional involvement of the frontoparietal regions. We report a case of a 10 year old girl who arrived at the pediatric emergency department with generalized swelling, dark-colored urine, and two days of seizures following recent throat infection. Her blood pressure consistently exceeded the 95th percentile by +12 mm Hg, indicating stage 2 hypertension. A computed tomography (CT) scan showed hypodensities (edema) in the parieto-occipital white matter, consistent with PRES. Due to limited resources, magnetic resonance imaging (MRI) could not be performed. The patient was treated symptomatically with levetiracetam for seizures and furosemide and amlodipine for hypertension. By the fifth day of hospitalization, the patient experienced significant improvement, with a return to normal appetite, urine color, and neurological function. Early diagnosis contributed to her full recovery. Physicians in resource limited settings should have high degree of suspicion of pediatric PRES and perform detailed history taking, examination, laboratory investigations and imaging (whenever available) for management of pediatric PRES.

## 1. Introduction

Posterior reversible encephalopathy syndrome (PRES) is a well-recognized condition that presents with altered mentation, seizures, visual loss, and white matter vasogenic edema involving occipital and parietal lobes with less commonly frontoparietal lobes [1]. It may develop at any age from infants to the elderly, but most frequently affects young or middle-aged adults, with a mean age of 45 years [2]. The incidence of PRES in the general pediatric population is 0.04%, 0.7% in children with cancer, and 0.4% in children with admission to the pediatric intensive care unit (PICU) [3].

Pediatric PRES has been reported in children with underlying disorders, renal diseases, systemic lupus erythematosus (SLE), sickle cell disease, bone marrow transplantation, use of chemotherapeutics [4, 5]. There have been only a few cases documenting the association between poststreptococcal glomerulonephritis (PSGN) and PRES [6]. The name ‘reversible' derives from the fact that both neurological and neuroimaging findings spontaneously recover within a few hours or 7–8 days after the initiation of treatment [7]. The intracranial hypertension, endothelial injury, and disruption of blood brain barrier are implicated in the pathogenesis [8]. Understanding the association between these two conditions may lead to earlier diagnosis and treatment of PRES, ultimately improving patient outcomes [6].

Although PRES is being increasingly diagnosed in adults, it is often overlooked in children by the primary physicians in most cases leading to delayed diagnosis, there is limited literature on pediatric PRES from resource limited settings where neuroimaging options like MRI are limited placing challenges pediatricians in accurately identifying and managing the condition.

Adhering the Case Report (CARE) guidelines, we present a case of a 10 year old girl diagnosed with PRES who made a full recovery after a 5 day hospital stay.

## 2. Case Report

Our patient, a 10 year old girl with no significant prior medical history, was brought to the pediatric emergency department with generalized swelling, cola-colored urine, and seizures for two days. Prior to these symptoms, she had an upper respiratory tract infection a few weeks earlier, treated with antibiotics from a local pharmacist, with symptoms resolving after the medication. On examination, her blood pressure (BP) remained persistently above the 95th percentile +12 mm Hg, indicating stage 2 hypertension. Bilateral pitting edema was observed on her lower limbs, and her neurological assessment revealed a Glasgow Coma Scale (GCS) score of 13/15, with no additional abnormalities noted, including no papilledema on fundus examination.

Urine analysis showed protein at 3+, red blood cells (RBC) at 8–10 hpf, and white blood cells (WBC) at 4–10 hpf. Her metabolic profile indicated blood urea of 30 mg/dL, creatinine of 0.7 mg/dL, sodium of 141 mEq/L, and potassium of 3.8 mEq/L. Serum C3 levels were low (0.58 mg/dL, reference range: 90–180 mg/dL), and her ASO titer was elevated to 800 IU/mL (reference range: < 200 IU/mL). Hematology results showed mild anemia (Hb 9.6 g/dL), leukocytosis (18,800), and normal platelet count (3.93 lacs).

**Figure 1:**
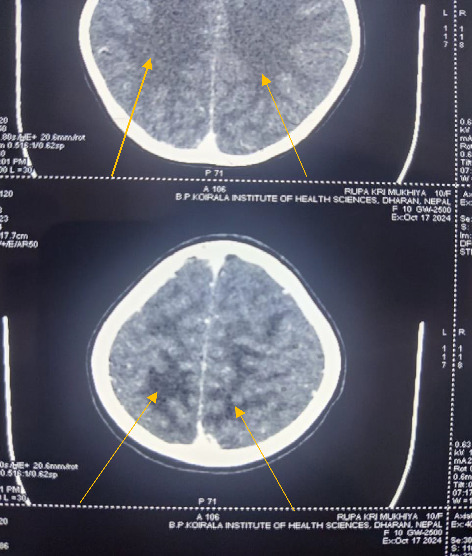
Computed tomography (CT) scan of the head region showing bilateral hypodensities (edema) in the parieto-occipital white matter (indicated by yellow arrow).

Levetiracetam was started at 20 mg/kg/day for seizure management, alongside furosemide at 2 mg/kg/day, and amlodipine at 0.1 mg/kg/day. Ceftriaxone, initially administered for suspected throat infection, was later discontinued after blood, urine, and Cerebrospinal Fluid (CSF) cultures were negative. On day 3, the patient experienced three brief, generalized tonic seizures, for which levetiracetam dosage was increased to 60 mg/kg/day. Given her persistently elevated BP (above the 95th percentile plus 12 mm Hg), she received labetalol (20 mg i.v. Bolus, followed by an infusion at 0.1 mg/kg/hr up to 0.7 mg/kg/hr) and an increased dose of amlodipine to 10 mg/day. The labetalol infusion was gradually tapered and discontinued over 36 h as her BP stabilized near the 50th percentile. A head computed tomography (CT) scan revealed bilateral hypodensities in the parieto-occipital white matter, indicative of edema consistent with PRES (Figure 1).

By the fifth day of hospitalization, the patient showed marked improvement, with a return of normal appetite, normal urine color, and neurological exam. She recovered fully with timely diagnosis and intervention, and no neurological consultation was required. She remains asymptomatic at her 20 day follow-up.

## 3. Discussion

PRES was diagnosed in this case as a result of hypertensive encephalopathy caused by acute PSGN, based on clinical symptoms, lab results, and imaging findings. PRES is uncommon in children, though its true incidence may be underreported due to a generally low diagnostic suspicion [5]. This clinical and radiological syndrome is marked by symptoms like headache, changes in mental status, seizures, and visual disturbances, often linked to hypertension [6].

Around 70%–80% of patients with PRES exhibit moderate to severe arterial hypertension [5]. The exact pathophysiology of PRES remains unclear, but it is thought to involve a disruption of the blood-brain barrier, leading to vasogenic edema primarily in the posterior areas of the brain. Contributing factors to PRES may include hypertension, renal disease, autoimmune disorders, and specific medications [6]. While hypertensive encephalopathy is a prevalent cause of PRES, it is rare for acute PSGN to result in hypertensive encephalopathy [9]. The timeframe for lesions to transition to an irreversible state is not clearly defined. Although seizures linked to severe hypertension may suggest hypertensive encephalopathy, they are among the most common symptoms of PRES. Typically, seizures are generalized; however, as demonstrated in this case, they can start focally and then generalize, potentially recurring and leading some patients to develop status epilepticus [9].

PRES is managed by early diagnosis [10], addressing symptoms and treating the underlying cause. A comparable case involved an 8 year old boy with new-onset seizures, followed by symptoms like headaches, blurred vision, fever, vomiting, and nausea. Symptomatic treatment included midazolam, lorazepam, levetiracetam, and antihypertensive medications (amlodipine, labetalol, and nicardipine). Further history revealed a recent respiratory infection and dark urine. MRI confirmed PRES. Adjusted medications stabilized his BP, and he was discharged on day six with stable renal function and normal BP [8]. Promptly identifying and eliminating the trigger or addressing the underlying condition is crucial for effective treatment [11]. During hypertensive crises, it is important to reduce BP by no more than 20%–25% in the initial hours to avoid cerebral, coronary, and renal ischemia [12]. Symptoms of PRES are frequently reversible, but the prognosis is influenced by the underlying conditions. Serious complications such as ischemic infarction, hemorrhage, or status epilepticus can lead to permanent deficits or death [11]. It has been reported that PRES recurs in 8% of patients following complete recovery from the initial episode [13].

Fatalities are typically associated with neurological complications and delays in diagnosis and treatment [14]. Recent reports questioning the reversible nature of PRES indicate that permanent neurological impairment can occur, with mortality rates reaching 15% [15].

## 4. Conclusion

PRES in children can often be overlooked by physicians and radiologists. Physicians should consider PRES as a differential diagnosis by carefully evaluating the patient's history to search for PRES-inducing disorders relevant to age group. This is particularly important in resource-limited settings where MRI is unavailable, and CT is the primary imaging tool like ours to avoid the possible complications and irreversibility of the disease.

## Data Availability

Data supporting the conclusions of this report are contained within the report. Additional nonrelevant patient data are protected under patient privacy regulations and policies.
